# Endoscopic ultrasound-guided embolization of a gastric varix by injection of RADA16 self-assembling peptide and coils

**DOI:** 10.1055/a-2261-7485

**Published:** 2024-03-14

**Authors:** Jonathan Wilen, Judy A Trieu, Todd H. Baron

**Affiliations:** 1 Gastroenterology and Hepatology, University of North Carolina, Chapel Hill, United States; 2 7548 Gastroenterology, Washington University in St Louis, St Louis, United States

Gastric variceal bleeding accounts for 10%–30% of variceal bleeding and has higher mortality than esophageal variceal bleeding 1 . Endoscopic ultrasound (EUS) offers new modalities for treatment and prevention of gastric variceal bleeding. Acrylate polymers such as cyanoacrylate are traditionally injected into gastric varices under EUS guidance. However, adverse events, such as systemic embolization, ulceration with rebleeding, and failed withdrawal of the needle, are drawbacks to cyanoacrylate use. Coil embolization is more effective with fewer adverse events than cyanoacrylate injection and with high technical and clinical success 2 . The combination of cyanoacrylate and coil embolization appears more effective than monotherapy 3 . Because of the particular handling and side-effect profile of cyanoacrylate, alternative agents have been used for treatment of gastric varices 4 . We present the use of a novel hemostatic gel (PuraStat; 3-D Matrix, Newton, Massachusetts, USA) injected for treatment of gastric variceal bleeding. PuraStat is composed of 2.5% RADA16, a synthetic hemostatic material that forms a 3D matrix mimicking human extracellular matrix 5 . It has been used in a variety of clinical scenarios with promising results 5 .

A 70-year-old woman with chronic portal vein thrombosis and noncirrhotic portal hypertension, who was taking apixaban, suffered recurrent gastric variceal bleeding that had previously required balloon-occluded retrograde transvenous obliteration and cyanoacrylate and coil embolization. The patient now presented with melena and a large gastric varix of 10 mm in diameter. Using an oblique echoendoscope, a standard 19G fine-needle aspiration device was used to place three embolization coils (Nester; Cook Medical) into the varix followed by injection of 3 mL of PuraStat ( Video 1 ). There was near complete obliteration of the varix as assessed by doppler imaging. The patient tolerated the procedure well with no adverse event and was discharged with planned surveillance for gastric varices. This is a novel use of a new hemostatic gel for the treatment of gastric variceal bleeding in conjunction with coil embolization. Further experience is needed to validate its use.

Use of a novel hemostatic gel in conjunction with coil embolization for the treatment of actively bleeding gastric varices.Video 1

Endoscopy_UCTN_Code_TTT_1AO_2AD

## Notes

Endoscopy E-Videos https://eref.thieme.de/e-videos
*E-Videos* is an open access online section of the journal *Endoscopy* , reporting on interesting cases and new techniques in gastroenterological endoscopy. All papers include a high-quality video and are published with a Creative Commons CC-BY license. Endoscopy E-Videos qualify for HINARI discounts and waivers and eligibility is automatically checked during the submission process. We grant 100% waivers to articles whose corresponding authors are based in Group A countries and 50% waivers to those who are based in Group B countries as classified by Research4Life (see: https://www.research4life.org/access/eligibility/ ). This section has its own submission website at https://mc.manuscriptcentral.com/e-videos .
